# Genome-Wide Analysis of the YABBY Transcription Factor Family in Pineapple and Functional Identification of *AcYABBY4* Involvement in Salt Stress

**DOI:** 10.3390/ijms20235863

**Published:** 2019-11-22

**Authors:** Zeyun Li, Gang Li, Mingxing Cai, Samaranayaka V.G.N. Priyadarshani, Mohammad Aslam, Qiao Zhou, Xiaoyi Huang, Xiaomei Wang, Yeqiang Liu, Yuan Qin

**Affiliations:** 1 Key Laboratory of Genetics, Breeding and Multiple Utilization of Crops, Ministry of Education, Fujian Provincial Key Laboratory of Haixia Applied Plant Systems Biology; State Key Laboratory of Ecological Pest Control for Fujian and Taiwan Crops, College of Life Sciences, Fujian Agriculture and Forestry University, Fuzhou 350002, China; lizeyun0514@163.com (Z.L.); caimingxing510704@163.com (M.C.); aslampmb1@gmail.com (M.A.); zhouqiao0606@163.com (Q.Z.); xiaoyi0922@163.com (X.H.); 2 College of Agriculture, Fujian Agriculture and Forestry University, Fuzhou 350002, China; whu_ligang@whu.edu.cn; 3 State Key Laboratory for Conservation and Utilization of Subtropical Agro-Bioresources, Guangxi Key Lab of Sugarcane Biology, College of Agriculture, Guangxi University, Nanning 530004, China; lyq91158@163.com; 4 Horticulture Research Institute, Guangxi Academy of Agricultural Sciences, Nanning Investigation Station of South Subtropical Fruit Trees, Ministry of Agriculture, Nanning 530007, China; wangxiaomei159@163.com

**Keywords:** YABBY, pineapple, expression pattern, subcellular localization, abiotic stress

## Abstract

The plant-specific transcription factor gene family, YABBY, belongs to the subfamily of zinc finger protein superfamily and plays an essential regulatory role in lateral organ development. In this study, nine *YABBY* genes were identified in the pineapple genome. Seven of them were located on seven different chromosomes and the remaining two were located on scaffold 1235. Through protein structure prediction and protein multiple sequence alignment, we found that *AcYABBY3*, *AcYABBY5 and AcYABBY7* lack a C2 structure in their N-terminal C2C2 zinc finger protein structure. Analysis of the *cis*-acting element indicated that all the seven pineapple *YABBY* genes contain multiple MYB and MYC elements. Further, the expression patterns analysis using the RNA-seq data of different pineapple tissues indicated that different *AcYABBYs* are preferentially expressed in various tissues. RT-qPCR showed that the expression of *AcYABBY2*, *AcYABBY3*, *AcYABBY6 and AcYABBY7* were highly sensitive to abiotic stresses. Subcellular localization in pineapple protoplasts, tobacco leaves and *Arabidopsis* roots showed that all the seven pineapple YABBY proteins were nucleus localized. Overexpression of *AcYABBY4* in *Arabidopsis* resulted in short root under NaCl treatment, indicating a negative regulatory role of *AcYABBY4* in plant resistance to salt stress. This study provides valuable information for the classification of pineapple *AcYABBY* genes and established a basis for further research on the functions of AcYABBY proteins in plant development and environmental stress response.

## 1. Introduction

Plants are often exposed to extreme environments during their development and growth. Abiotic stresses such as salt, drought, high temperature and cold lead to adverse effect on growth and development of plants, resulting in yield loss. In plants, a series of defense systems play a vital role in survival under extreme external environmental changes. Transcription factors play a crucial role in plant defense system regulating gene expression, some of which are associated with the abiotic stress response [1].

A number of transcription factors are plant-specific. Structurally, transcription factors are divided into four functional regions, namely DNA binding domain, oligomeric site, transcriptional regulatory domain and nuclear localization signal region. These functional domains determine the characteristics, function, regulation and nuclear localization of transcription factors. The YABBY transcription factor family is widely present in plants and is a subfamily of the zinc finger protein superfamily. YABBY transcription factor possess two conserved domains, the N-terminal zinc finger domain and the C-terminal YABBY domain. The amino acid residues in these two domains are highly conserved and these domains are involved in the specific binding of DNA [2].

The evolutionary history of *YABBY* gene family is consistent with the origin of the leaves of seed plants. These transcription factors are specific to seed plants [3] and play important regulatory roles in the development of plants lateral organ. There are five YABBY subfamilies in angiosperms, namely *INO*, *CRC*, *YABBY2*, *FIL*/*YABBY3* and *YABBY5* [4]. The *YABBY* genes are well studied in *Arabidopsis thaliana*, where the six members of *YABBY* genes revealed overlapping functions [2,5]. All *AtYABBYs* promote the differentiation of the abaxial surface cells of lateral organs and participate in the establishment of dorsal-ventral polarity, leaf expansion and flower organ development [6,7]. Although they display similar functions, yet different *AtYABBY* genes have diversified expression pattern and function. For example, *AtYABBY2*, *AtYABBY3* and *AtFIL* genes are specifically expressed in the distal region of the aerial part of plants and *Arabidopsis* overexpression of *AtYABBY3* and *AtFIL* exhibited leaf-rolling [8,9]. *AtYABBY2*, *AtYABBY3*, *AtFIL* and *AtYABBY5* show functional redundancy during leaf development [10]. While *AtCRC* regulates the development of carpel and nectary [3,10]. *AtINO* mainly regulates the development of ovules [11]. In rice, *DL* and *OsYABBY1*, the homologs of *AtYAB2* and *AtCRC* are not expressed in a polar manner in the lateral organs and their functions are not associated with polarity regulation of lateral organ development [12,13]. *OsDL* mainly regulates the carpel identity and the formation of main veins of the leaves by promoting cell proliferation in the central region of rice leaves [14,15] whereas, *OsYABBY1* was found to be involved in the feedback regulation of gibberellin (GA) biosynthesis and metabolism resulting semi-dwarf phenotype in overexpression lines. [16,17]. Although *OsYABBY3* is involved in leaf development, unlike its function in *Arabidopsis*, it does not affect the establishment of leaf polarity [18]. Other than above developmental processes, the YABBY family *Shattering1* gene is responsible for seed shattering in cereals including sorghum, rice and maize [19].

Pineapple (*Ananas comosus* L.), a tropical edible fruit, is a perennial monocotyledon belonging to the Bromeliaceae. The completed assembly of the whole genome of pineapple [20] provides an opportunity for the systematic study of the pineapple YABBY family. Here, we identified 9 *YABBY* genes in pineapple and analyzed the gene structure, motif pattern of AcYABBYs, phylogenetic relationship of YABBYs between pineapple, *Arabidopsis* and rice. We found 7 *AcYABBY* genes located on seven different chromosomes. Through protein structure prediction and protein multiple sequence alignment analysis, we discovered that AcYABBY3, AcYABBY5 and AcYABBY7 lack a C2 at the N-terminus and thus could not constitute a C2C2 zinc finger domain. We further performed subcellular localization analysis for the seven YABBY proteins in pineapple protoplasts, tobacco leaves and *Arabidopsis* roots and found that the AcYABBY proteins were mainly localized in the nucleus. Moreover, we also analyzed the promoter *cis*-acting element and found that all the seven *AcYABBYs* have MYB and MYC domains, those are involved in drought, low temperature, salt and ABA stress responses. We further performed RT-qPCR analysis and showed that the expression levels of the seven *AcYABBYs* were changed under different abiotic stresses, including salt, drought, cold, hot, ABA and ethephon stresses at different time points. Finally, we found that over-expression of *AcYABBY4* in *Arabidopsis* resulted in increased susceptibility to salt stress. This study provides comprehensive information about pineapple AcYABBY proteins and a basis for studying the function of *AcYABBY* family members in plant development and response to environmental stresses.

## 2. Results

### 2.1. Identification and Characterization of the Pineapple YABBY Transcription Factors

**Figure 1:**
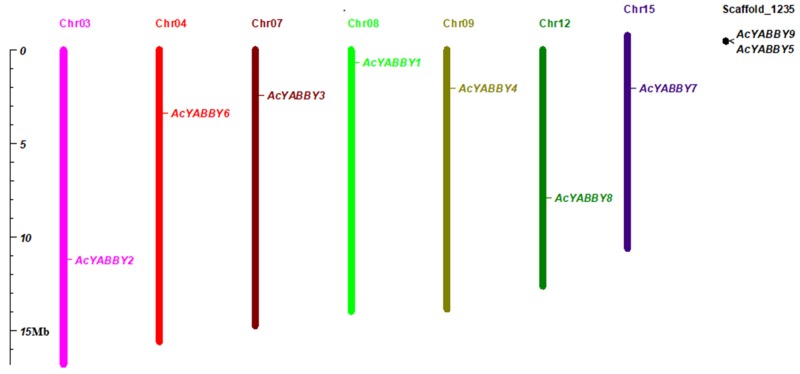
Distribution of YABBY genes in pineapple genome. Pineapple chromosomes and scaffolds having YABBY genes are shown here. The length of bar represents the size of the chromosome.

To identify *AcYABBY* genes, BLAST and Hidden Markov Model searches were used to search the pineapple genome with *YABBY* sequences from *Arabidopsis* as query. A total of 9 *AcYABBY* genes were identified from pineapple genome and named *AcYABBY1* to *AcYABBY9*. The protein lengths of these genes ranged from 49 aa (*AcYABBY8*) to 226 aa (*AcYABBY2*) with the corresponding molecular weight ranging from 5383.98 to 24706.13 Da. The additional information about *AcYABBYs* transcript ID, gene name, proteins size, protein isoelectric point and exons are listed in Table 1. There are two genes, *AcYABBY8* and *AcYABBY9*, with incomplete N-terminal sequence. We found that the CDSs of *AcYABBY8* and *AcYABBY9* lack ATG, which codes the initiation codon, suggesting we did not obtain the full length genes. Unfortunately, the conserved YABBY domain was located at the N terminal of YABBY proteins according to the reported studies and our conversation assay. To obtain more exact results, we considered the remaining seven genes for further studies. According to the mapping results, seven *AcYABBY* genes were localized on seven different chromosomes and two genes were located on scaffold1235 (Figure 1). 

### 2.2. Phylogenetic Analysis of YABBY Family Genes

**Figure 2:**
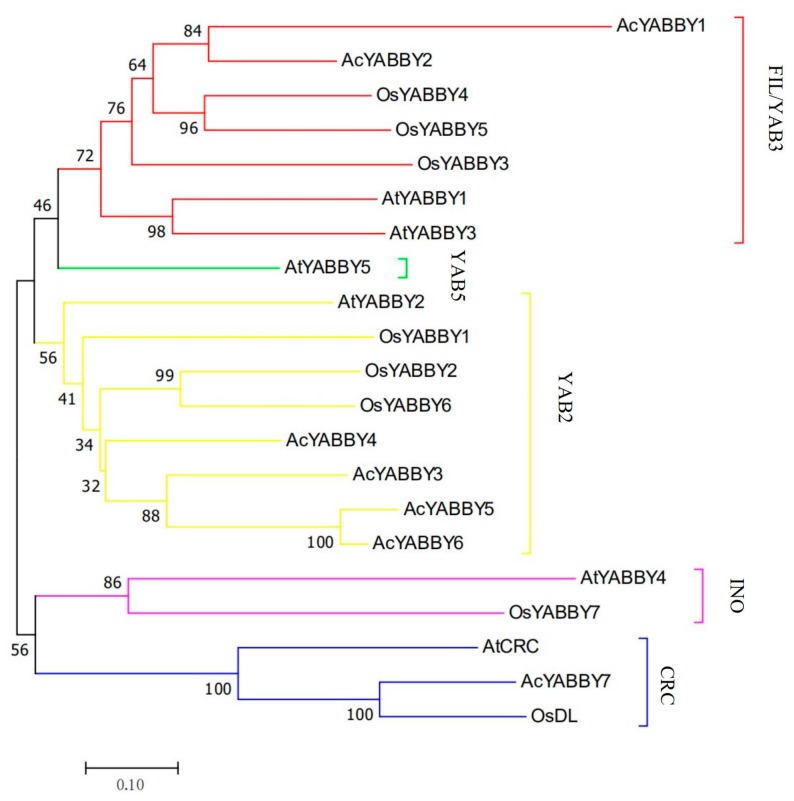
Phylogenetic relationship of the *YABBYs* proteins of Ac (Pineapple), At (*Arabidopsis thaliana*), Os (Oryza sativa). The phylogenetic tree was made using neighbor-joining with a bootstrap values of 1000. Phylogenetic tree divides *YABBYs* into five subfamilies (*YAB2*, *CRC*, *YAB5*, *FIL*/*YAB3 and INO*). Each subfamily is represented with different color.

**Figure 3:**
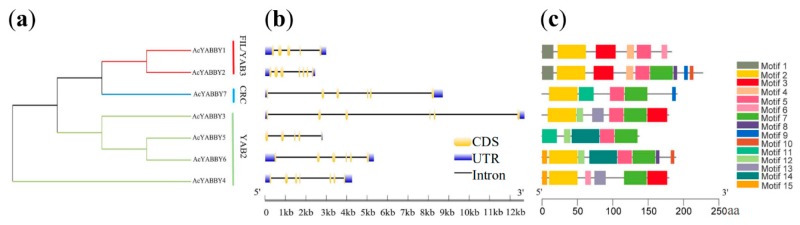
Phylogenetic relationships, gene structure and architecture of conserved protein motifs in pineapple YABBY genes. (**a**) The phylogenetic tree was constructed based on the full-length sequences of pineapple YABBY proteins using MEGA 7 software. Details of clusters are shown in different colors. (**b**) Exon-intron structure of pineapple YABBY genes. Blue boxes represent untranslated 5′- and 3′-regions; yellow boxes represent exons; black lines represent introns. (**c**) The motif composition of pineapple YABBY proteins. The motifs, numbers 1–15, are displayed with different colored boxes.

A phylogenetic tree was constructed to determine the phylogenetic relationships of *YABBY* genes in pineapple, *Arabidopsis* and rice. Phylogenetic analysis of 7 pineapple *YABBY* genes, 6 *Arabidopsis YABBY* genes and 8 rice *YABBY* genes was performed by generating a neighbor-joining phylogenetic tree (Figure 2). The result showed that the *YABBY* genes of these three species could be divided into five subfamilies—YAB2, CRC, YAB5, FIL/YAB3 and INO. However, the *AcYABBYs* were divided to three subfamilies—FIL/YAB3, CRC and YAB2 (Figure 3a). Among these subfamilies, YAB2 had the largest members with four pineapple genes, one *Arabidopsis* gene and three rice genes. FIL/YAB3 was the second largest subgroup, containing two pineapple genes, two *Arabidopsis* genes and three rice genes. CRC contained three genes, including one pineapple gene (*AcYABBY7*), one *Arabidopsis thaliana* gene (*AtCRC*) and one rice gene (*OsDL*). However, INO contained one *Arabidopsis* gene (*AtYABBY4*) and one rice gene (*OsYABBY7*), yet no pineapple gene was categorized into this group. The smallest subgroup, YAB5, only had one *Arabidopsis* gene (*AtYABBY5*), suggesting that YAB5 may have a particular function in the *Arabidopsis thaliana*.

### 2.3. Gene Structure Analysis and Identification of Conserved Motifs

To investigate the structure diversity, we used Gene Structure Display Server [21]. The results showed that the exon number of the *AcYABBY* genes ranged from a minimum of 2 and a maximum of 7. *AcYABBY2* and *AcYABBY7* had the maximum exons numbers (Figure 3b). The schematic diagram representing the structure of AcYABBY proteins was constructed from the results of the MEME motif analysis (Figure 3c). The number of motifs ranged from 1 to 15 [22]. *AcYABBY5* and *AcYABBY7* contained five motifs. *AcYABBY1, AcYABBY3* and *AcYABBY4* had six motifs, while *AcYABBY6* had eight motifs. *AcYABBY2* had the largest number of motifs, containing nine motifs. The similar motif arrangements among AcYABBY proteins indicate that the protein architecture is conserved within a particular subfamily. The functions of these conserved motifs remain to be elucidated. 

### 2.4. AcYABBY Protein Homology Modeling and Sequence Alignment

**Figure 4:**
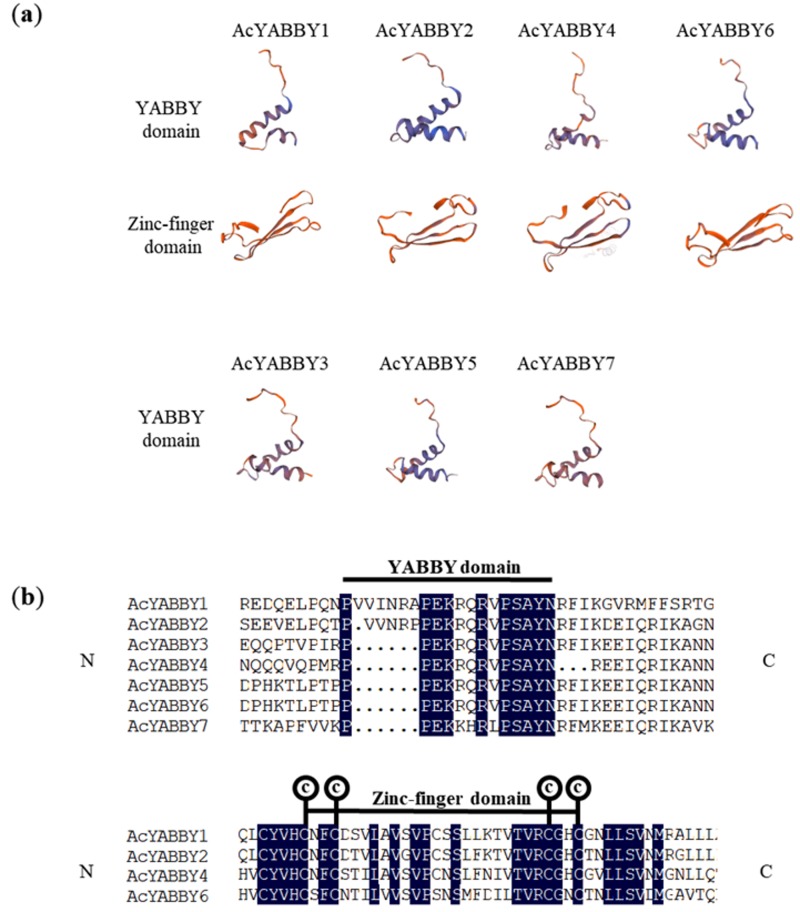
Predicated structures and multiple sequence alignment of pineapple YABBY proteins. (**a**) Predicted protein structures of AcYABBY proteins. (**b**) Multiple sequence alignment of AcYABBYs, zinc-finger domain and YABBY domain amino. ′N′ and ′C′ indicate the N-terminal and C-terminal. The four cysteine residues putatively responsible of the zinc-finger structure are also indicated. Identical amino acids are highlighted in blue.

The YABBY family possesses a C2C2 zinc finger domain at the N-terminus and a YABBY domain at the C-terminus. To study AcYABBY protein conformation, seven pineapple YABBY protein were analyzed by SWISS-MODEL to identify the best template with known structures and similar sequence. All the seven AcYABBY proteins were predicted with a YABBY domain. Whereas, only four AcYABBY proteins contained a zinc-finger domain (Figure 4a). The zinc-finger domain was not detected in the three *AcYABBY* genes (*AcYABBY3*, *AcYABBY5*, *AcYABBY7*). To analyze further, we used DNAMAN for domain analysis of protein sequence alignment (Figure 4b). We found that one C2 structure was missing from the C2C2 structure in the protein sequences of the these three *AcYABBY* genes. Therefore, we concluded that zinc finger structure was incomplete and unpredictable in *AcYABBY3*, *AcYABBY5*, *AcYABBY7*. The specific cause of this phenomenon is still unclear and may be related to the evolution of the pineapple *YABBY* genes.

### 2.5. Expression Profiling and Subcellular Localization of AcYABBYs

**Figure 5:**
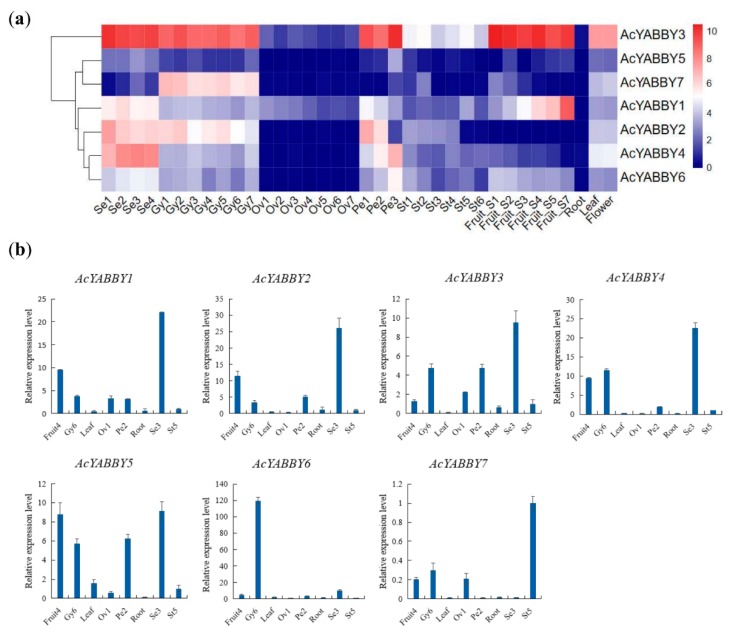
Expression profiles of the pineapple *YABBY* genes. (**a**) Heat-map of *AcYABBY* genes expression profiles in different tissue generated from RNA-seq data. The *AcYABBYs* were clustered according to their expression patterns. Red color indicates high levels of transcript abundance and blue indicates low transcript abundance. The color scale is shown on the right. Samples are mentioned at the bottom of each lane: Se (sepal) Se1-Se4, Gy (gynoecium) Gy1-7 Ov (ovule) Ov1-Ov7, Pe (petal) Pe1-Pe3, St (stamen) St1-St5, Fruit S1-S7, Root, Leaf, Flower. (**b**) Expression analysis of 7 pineapple *YABBY* genes in eight representative samples by RT-qPCR. RT-qPCR data were normalized using pineapple *PP2A* gene and vertical bars indicate standard deviation.

**Figure 6:**
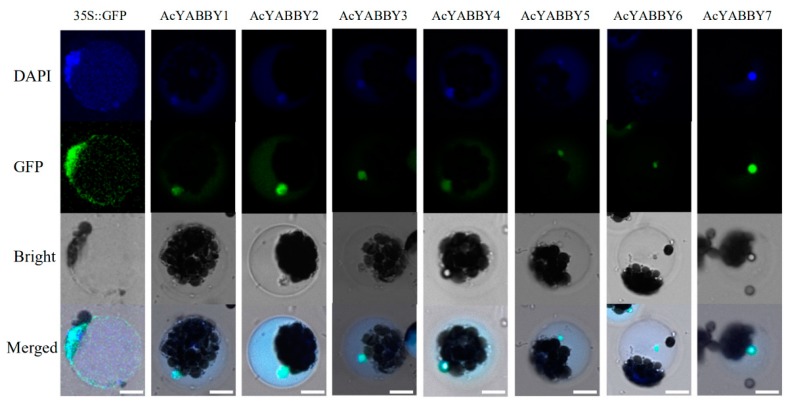
Subcellular localization of pineapple YABBY proteins in pineapple protoplasts. The *35S::AcYABBYs-GFP* and *35S::GFP* control vectors were transiently expressed in pineapple protoplasts. Results were visualized by a confocal microscope after 16 h transformation. Bar = 10 μm.

Transcriptome data from different developmental stages of pineapple tissue were used to study the expression patterns of seven *AcYABBY* genes to understand the transcriptional diversity of the *AcYABBY* genes. The expression levels of *AcYABBY* genes were analyzed by FPKM values from RNA sequence data. Hierarchical clusters and expression patterns of each gene were generated based on the average log values of each gene in each tissue. According to Heat-map analysis, the *AcYABBY3* expressed ubiquitously in most organs. The *AcYABBY3* was highly and specifically expressed at all stages of development of the organs (sepal Se1-4, gynoecium Gy1-7, petal Pe1-3, fruit S1-7, flower and leaf). Conversely, the *AcYABBY5* and *AcYABBY6* showed relatively low expression levels in almost all organs. Besides, some genes were highly expressed in specific organs. For example, *AcYABBY1* was highly expressed in sepal (Se2 stage) and fruit (S4-7 stages). *AcYABBY2* was highly expressed in sepal (Se1-4 stages), pistil (Gy1/2/4/5 stages) and petal (Pe1-2 stages). *AcYABBY4* was highly expressed in sepal (Se1-4 stages) and petal (Pe3 stage). *AcYABBY7* was highly expressed in gynoecium (Gy1-7 stages) (Figure 5a). The reliability of transcriptome data was further verified by RT-qPCR experiment, which was carried out on eight representative samples for seven *YABBY* genes. The results revealed that expression patterns of the *YABBY* genes detected by RT-qPCR were partially consistent with the results of RNA-seq analysis (Figure 5b). The differences between RT-qPCR and RNA-seq may be caused by sampling and it is difficult to keep the samples’ stage exactly at the same as RNA-seq sequencing samples. To understand molecular characteristics of *AcYABBYs*, seven pineapple *YABBY* genes (*YABBY1-7*) were selected for subcellular localization. In pineapple protoplasts, *AcYABBYs-GFP* were co-localized with a DAPI signal, suggesting that the AcYABBY proteins were mainly localized in the nucleus (Figure 6). Also, the *AcYABBYs-GFP* fusion proteins were also localized in the nucleus of tobacco leaves (Figure S1) and *Arabidopsis* roots (Figure S2). These localization results were consistent with each other, suggesting that AcYABBY proteins were localized to the nucleus.

### 2.6. Cis-Acting Elements and RT-qPCR Analysis of AcYABBY Ggenes

**Figure 7:**
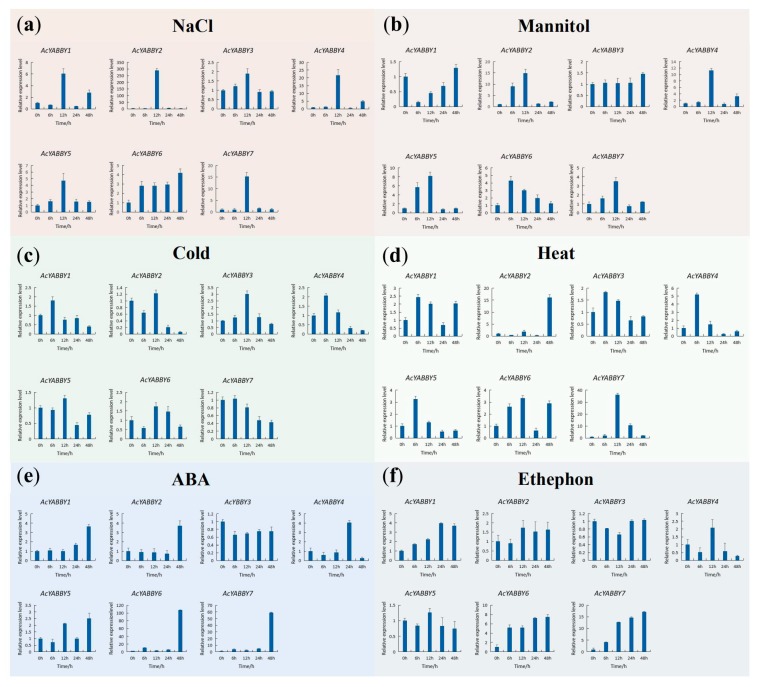
Expression profiles of 7 selected *AcYABBY* genes in response to various abiotic stress treatments. (**a**) NaCl treatment. (**b**) Mannitol treatment. (**c**) Cold treatment. (**d**) Heat treatment. (**e**) ABA treatment. (**f**) Ethephon treatment. RT-qPCR data were normalized using pineapple *PP2A* gene as reference gene. Error bars indicate Standard Deviation.

The study of the *cis*-element indicated that each of the pineapple genes comprised the MYB and MYC elements in their promoter regions. MYB elements have been reported to be associated with drought, low temperature, salt and ABA stress responses and MYC elements are associated with drought and salt stress response [1]. Besides, the majority of the pineapple *YABBYs* except *AcYABBT3, AcYABBT4 and AcYABBT7* also contained at least an ERE element, which responds to ethylene. The identified Motif of the *cis*-acting elements and the sequences are listed in Table 2. To investigate the expression pattern of *AcYABBY* genes in response to different abiotic stresses, RT-qPCR was carried out using pineapple leaves exposed to various abiotic stresses for different time intervals such as 0, 6, 12, 24 and 48 h (h). The *AcYABBY* genes expressed diversely under the six types of abiotic stresses. Under salt stress (150 mM NaCl), the transcription level of *AcYABBYs* increased gradually. Among them, *AcYABBY1*, *AcYABBY2*, *AcYABBY4*, *AcYABBY5* and *AcYABBY7* transcript levels were highly up-regulated with the highest expression at 12 h. Notably, *AcYABBY2* increased to a peak (~250-fold) at 12 h and then rapidly declined to a level similar to the control (Figure 7a). Under drought stress, *AcYABBY2* increased to a peak of ~15-fold at 12 h. *AcYABBY4* increased to a maximum of ~10-fold at 12 h. *AcYABBY5* increased to a maximum of ~8-fold at 12 h. After 12 h, the expression level of *AcYABBY2*, *AcYABBY4 and AcYABBY5* rapidly declined to a level similar to the control when plants were subjected to drought stress (Figure 7b). Expression level of *AcYABBY*3 increased slowly to a peak of ~3-fold at 12 h and then declined to a level similar to the control under cold stress (Figure 7c). Under heat stress, *AcYABBY7* increased to a peak of ~30-fold at 12 h and then rapidly declined to a level similar to the control. *AcYABBY2* rose to a maximum of ~15-fold at 48 h (Figure 7d). Under ABA stress, *AcYABBY6* and *AcYABBY7* transcript levels were highly up-regulated at 48h. *AcYABBY6* increased to a peak of ~100-fold and *AcYABBY7* increased to a maximum of ~60-fold at 48h (Figure 7e). Under ethephon stress, *AcYABBY6* increased to a maximum of ~8-fold at 48h and *AcYABBY7* increased to a maximum of ~17-fold at 48h (Figure 7f). These results together suggested that *AcYABBYs* are involved in plant response to abiotic stresses. 

### 2.7. AcYABBY4 Negatively Regulates the High Salinity Tolerance in Arabidopsis

**Figure 8:**
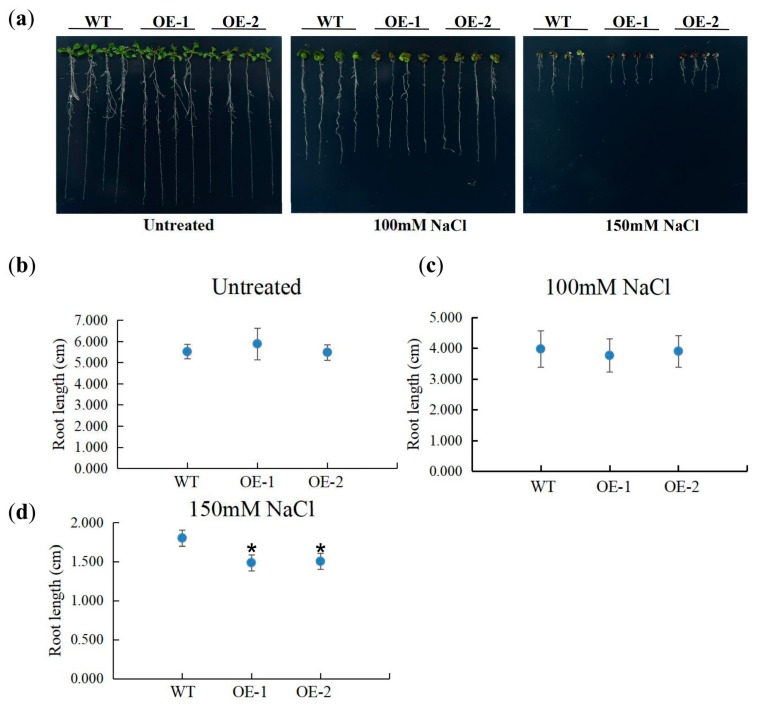
Phenotypic comparison of wild-type (WT) and *AcYABBY-over-exression* (*AcYABBY-OE*) *Arabidopsis* seedling under salt treatment. (**a**) Seedlings grown on ½ MS medium for three days were transferred to ½ MS medium supplemented with NaCl or without NaCl and allowed to grow for additional seven days. (**b**) Comparative root lengths of WT and transgenic lines in control conditions. (**c**) Comparative root length between WT and transgenic lines under 100mM NaCl treatment. (**d**) Comparative root lengths of WT and transgenic lines under 150 mM NaCl treatment. * denotes the significant difference (* *p* < 0.05) of t-tests between the transgenic lines and wild type.

To further investigate the function of *AcYABBYs* upon abiotic stress, we overexpressed *AcYABBY4* driven by 35S promoter in *Arabidopsis* plants and compared the growth phenotype of *AcYABBY4-overexpression (AcYABBY4-OE)* lines with the wild-type plants under optimum and salinity condition. For the phenotype comparison under salt stress, seedlings were cultured vertically on ½ MS medium for three days and then transferred to ½ MS medium supplemented with or without NaCl and allowed to grow for additional seven days. The root length of all lines showed a similar phenotype when grown on ½ MS medium without NaCl (Figure 8a,b). Under the 100 mM NaCl treatment, the root length of *AcYABBY4-OE* seedlings were also comparable to that of wild-type (Figure 8c). Whereas, under 150 mM NaCl treatment, the root length of *AcYABBY4-OE* seedlings were significantly reduced compared to wild-type seedlings (Figure 8d). Collectively, the results indicate that *AcYABBY4-OE* plants are susceptible to high salinity stress, suggesting that *AcYABBY4* may be a negative regulator in plant response to high salinity stress.

## 3. Discussion

### 3.1. Diversity of YABBY Transcription Factors in Plants

The plant-specific YABBY transcription factor family is involved in early embryonic development and lateral organ development. In particular, it plays an important role in the establishment of the near-distal axis polarity of leaves and is also involved in biological processes, such as plant development and stress response [23]. The *YABBY* genes are well studied in dicotyledonous plants such as *Arabidopsis.* However, relatively less information is available about monocot *YABBY* genes though it is reported in rice. In *Arabidopsis*, there are six members in YABBY family, namely *AtCRC*, *AtYABBY1*, *AtFIL*, *AtYABBY3*, *AtINO*/*AtYABBY4*, *AtABBY5* [2,5], with their unique and overlapping functions [17,24]. Various evidences suggest that they promote the differentiation of the distal axis of lateral organ cells [6,7] and have important effect on leaf expansion and floral organ development. In rice, there are eight *YABBY* members, *OsDL*, *OsYABBY1*, *OsYABBY2*, *OsYABBY3*, *OsYABBY4*, *OsYABBY5*, *OsYABBY6* and *OsYABBY7* [10]. They play important roles in regulating the development of lateral organs such as leaves and flowers [25]. Pineapple (*Ananas comosus* L.) is the third most economically important tropical fruit crop in the world after bananas [26]. Scientists are trying to improve its quality by improving resistance to environmental changes, increase the yield and improve its taste [27]. Therefore, the functional study of *YABBY* genes in regulation of plant development and stress response is important for breeding programs and agricultural production [25]. However, there are no reports on the characterization of pineapple YABBY proteins until now.

Here, we identified nine *YABBY* genes in pineapple genome and named them from *AcYABBY1* to *AcYABBY9*. According to phylogenetic analysis, pineapple *YABBY* genes were more closely related to rice *YABBY* genes, because pineapple as a perennial monocot is evolutionarily more related to grasses, including corn, rice and wheat. The YABBY transcription factor family is a subfamily of the zinc finger protein superfamily, with a zinc finger domain at the N-terminus and a “helix-loop-helix” YABBY domain at the C-terminus similar to HMG-box, which have been confirmed to be associated with specific binding of DNA [2]. Some of the pineapple YABBY proteins are highly conserved with *Arabidopsis* and rice YABBY proteins. We found that some pineapple *YABBY* genes, including *AcYABBY3*, *AcYABBY5* and *AcYABBY7*, lack a N-terminal C2C2 zinc finger domain. It could be due to a technical issue in the process of genome assembly. To have more exact results, we only selected seven *YABBY* genes for further studies with *YABBY* gene features.

### 3.2. AcYABBY Gene Expression Profiles Analysis

The completion of the pineapple genome sequence has provided us an opportunity to explore the specific genes responsible for specific traits [20]. As shown in Figure 5, hierarchical clusters and expression patterns shows that the *AcYABBY* genes have distinct expression pattern. For example, *AcYABBY7* is highly expressed in pistil. It was reported that *OsDL* is involved in the regulation of floral meristem [13]. Phylogenetic analysis indicate that *AcYABBY7* and *OsDL* are in the same subcluster, implying that *AcYABBY7* may also be involved in the tissue development of pineapple pistil. Interestingly, some *AcYABBYs* are preferentially expressed in floral organs. For example, *AcYABBY3* and *AcYABBY2* are enriched in sepals, pistils and petals. The specific enrichments of *AcYABBY4* in sepals and petals suggests its probable role in floral development of pineapple. *OsYABBY1* was found to be involved in the feedback regulation of gibberellin (GA) metabolism in rice [16]. Phylogenetic analysis shows that *AcYABBY3* and *AcYABBY4* and *OsYABBY1* are clustered together. It will be interesting to test whether *AcYABBY3* and *AcYABBY4* are also involved in the feedback regulation of gibberellin metabolism. Besides, we found that *AcYABBY3* and *AcYABBY1* are highly expressed in the fruit. It will be worth to investigate their potential role in the pineapple fruit development. The expression pattern of *AcYABBYs* and its corresponding function of rice homologous genes provide us a clue for understanding the function of *AcYABBYs* in pineapples.

### 3.3. AcYABBY4 Inhibits Root Growth of Seedlings under Salt Stress

Pineapple plant growth is affected by many factors during its growth and development, such as the uneven distribution of hormones, drought, salt and other adverse environmental conditions. In soybean, *YABBY* genes are involved in abiotic stress response and *GmYABBY10* act as a negative regulator of salt and drought stress in *Arabidopsis* [1]. However, the function of *AcYABBY* in plant response to abiotic stresses remains unknown. We analyzed the *cis*-acting elements of the pineapple YABBY family and found that the *AcYABBY* genes also comprise *cis*-acting elements such as MYB, MYC and ERE in their promoters. These *cis*-acting elements are involved in abiotic stress response in plants such as MYB elements are involved in salt, drought, low temperature and response to ABA [28,29]. MYC elements are involved in salt, drought and ABA stress response and ERE is related to ethylene response. Plant abiotic stress-responsive transcription factors could bind to these *cis*-acting elements [1]. The presence of these *cis*-elements in the promoter of *AcYABBYs* indicate that *AcYABBYs* are associated with stress response and may also be involved in plant adaptation to the environmental changes. Studying the function of the *YABBY* genes in plant response to abiotic stresses may help us to improve the agriculture production of pineapple. Using RT-qPCR we analyzed the response of the AcYABBY gene family under abiotic stress conditions such as NaCl, drought, cold, heat, ABA and ethephon treatments. The results showed that the expression patterns of pineapple *YABBY* genes were different under six stress conditions. For example, the expression of *AcYABBY2*, *AcYABBY3*, *AcYABBY4*, *AcYABBY6* and *AcYABBY7* was induced by NaCl, cold, drought, ABA and heat stress, respectively (Figure 7). These results suggest that *AcYABBYs* may play an important role in response to abiotic stress. The response of plants to abiotic stress is a complex process that is regulated by different molecular and cellular pathways. Here, the response of the *YABBY* genes to six different stresses laid the foundation for further functional study of the pineapple *YABBY* genes. To investigate the *AcYABBYs*’ functions, we constructed all the *YABBYs*’ over expression vectors and transformed it into *Arabidopsis*. We noted that the *AcYABBY4* over expression lines showed obvious phenotype with vegetative growth and development. Considering that the *AcYABBY4* basically had an early response than *AcYABBY2* during different stress conditions and the *AcYABBY4* indeed have a significant response to NaCl stress, so we chose *AcYABBY4* as our target gene for the functional analysis. However, *Arabidopsis* overexpressing *AcYABBY4* showed delayed growth and small seedlings size than wild-type (WT). We also found that the root growth of *AcYABBY4* overexpression lines were like WT and shows a normal phenotype in early stages of growth. Therefore, to avoid the effect of developmental changes on interpretation of our results of salt stress, we selected root growth as the parameter to study the salt stress. We found that the *Arabidopsis* overexpressing *AcYABBY4* is more sensitive to salt stress, indicating that *AcYABBY4* facilitates plants to perceive changes in the external salt environment more quickly and then inhibits plant growth through other complex regulatory mechanisms. Therefore, *AcYABBY4* may be a negative regulator of salt tolerance in transgenic *Arabidopsis* (Figure 8), providing a reliable basis for understanding *YABBY* participation in the biological process of plant stress response.

There is a close relationship between hormonal signal transduction and stress response. The response elements related to ABA and ethylene have important functions in plant abiotic stress and disease resistance. These observations also suggests that the *YABBY* genes may play an important role in response to ABA and ethylene accumulation of pineapple plants. The regulation mechanism of AcYABBY proteins under biological stress and in hormone signal transduction still remains elusive and could be a key part of further study of AcYABBYs. 

## 4. Materials and Methods

### 4.1. Identification of YABBY Transcription Factors in Pineapple

The pineapple YABBY protein and genome sequences were downloaded from Phytozome 12 (https://phytozome.jgi.doe.gov/pz/portal.html) and PlantTFDB (http://planttfdb.cbi.pku.edu.cn/) (Table S4). To identify the pineapple *YABBY* genes, HMMER3.02 (http://hmmer.wustl.edu/) with default parameters settings were used to search for the PFAM YABBY domain (PF04690) (http://pfam.sanger.ac.uk/). Also, BLAST (https://blast.ncbi.nlm.nih.gov/Blast.cgiwas) was used to search for potential pineapple genes containing the YABBY transcription factor. To achieve the accuracy of the analysis, we further analyzed the conserved sequence using NCBI-CDD (http://www.ncbi.nlm.nih.gov/Structure/cdd/wrpsb.cgi) with an E-value threshold of 0.013 and abandoned any sequences lacking the YABBY annotation [30]. The isoelectric point (pI) and molecular weight (MW) of the AcYABBY proteins were predicted using ExPASy-Compute pI/MW (https://web.expasy.org/compute_pi/).

### 4.2. Chromosome Locations of Pineapple YABBY Genes 

The information about the chromosome location of the *AcYABBY* genes were obtained from Phytozome. Based on the starting position of the gene and the length of the relevant chromosome, Mapchart [31] was used to visualize the localization of the pineapple *YABBY* gene on chromosomes and scaffolds.

### 4.3. Construction of Phylogenetic Tree

To generate phylogenetic trees, amino acid sequences of YABBYs in *Arabidopsis*, rice and pineapple were compared and analyzed by MEGA7 [32]. The protein sequences were aligned by MUSCLE and phylogenetic tree was constructed using Neighbor-Joining method with a bootstrap value of 1000.

### 4.4. Gene Structure Analysis and Identification of Conserved Motifs

The exon-intron substructure map of the pineapple *YABBY* genes were analyzed using online tool GSDS (http://gsds.cbi.pku.edu.cn/). The motifs of the AcYABBY proteins were determined using the MEME Suite 5.0.5 with classic mode, zoops for selecting the site distribution and 15 motifs. (http://meme-suite.org/).

### 4.5. Modeling of Protein 3D Structures and Sequence Alignment of AcYABBY Proteins

SWISS-MODEL (https://www.swissmodel.expasy.org) was used to predict the structures of seven YABBY proteins. The templates used in the study are listed in Table S1. The alignment of the AcYABBY protein sequences were carried out using DNAMAN [33].

### 4.6. RNA-Seq and RT-qPCR Data Analysis

To understand the expression pattern of *YABBY* genes in different development stages of pineapple, sepal, pistil, ovule, petal, stamen, fruit, root, leaf and flower RNA-Seq data (SRA315090) was downloaded from the NCBI database [20]. The trimmed pair-end reads of all tissues were aligned with the pineapple genome using TopHat v2.1.1 [34] with default parameter settings. The FPKM values were derived from Cuffdiff v2.2.1 and then a heatmap of *YABBY* genes expression was generated by the Rmap software package. To determine the relative transcript level of the selected *AcYABBY* genes, total RNA was extracted from different tissues (sepal, pistil, ovule, petal, stamen, fruit, root, leaf) of pineapple according to the procedure in the description of the RNA Plant Extraction Kit (OMEGA, Guangzhou, China). RNA quality was tested using gel electrophoresis and NanoDrop2000c (Thermo Fisher Scientific, Fujian, China). One μg of purified total RNA was reverse transcribed into cDNA in a 20 μL reaction volume using AMV reverse transcriptase (Takara, Beijing, China) according to the supplier′s instructions. Quantitative primers were designed by IDT (http://www.idtdna.com/pages/products/gene-expression/primetime-qpcr-assays-and-primers), with G+C content between 45%–55%, primer length between 17–25 bases, Tm value between 58–62 and quantitative product size between 80–150 bp. The reaction volume of RT-qPCR was 20 μL (10 μL 2×TransStart Top Green qPCR SuperMix, 8 μL nuclease-free water, 0.5 μL forward primer, 0.5 μL reverse primer and 1ul cDNA) according to the supplier′s instructions (TransGen Biotech, Beijing, China). The RT-qPCR parameters were as follows: 95 °C for 30 s; 95 °C for 5 s and 60 °C for 40 s for 40 cycles; 95 °C for 15 s. In each case, three technical replicates and at least three independent biological replicates were used. The primers used in this study are listed in Table S2. For RT-qPCR data analysis, the quantification cycle (Cq)value was automatically calculated by the Bio-Rad CFX Manager 3.1 system software and the delta-delta Cq method was used to calculate the relative expression levels. 

### 4.7. Vector Construction and Subcellular Localization

The full-length of coding sequences of the *AcYABBY* genes were amplified using the primers listed in Table S3. The PCR fragments were cloned into the pENTR/D-TOPO vectors (Invitrogen) and sequenced separately. The positive clones were recombined into the destination vector pGWB505 using LR reaction. The vectors harboring the *AcYABBY* genes were transformed into *Agrobacterium tumefaciens* (GV3101) and infiltrated to tobacco leaves. Subcellular localizations in tobacco leaves were observed with confocal microscope using GFP and DAPI stain. The *A. tumefaciens* (GV3101) with *AcYABBY* genes were also used to transform wild-type *Arabidopsis* by a floral dip procedure [35]. Transgenic *Arabidopsis* lines were selected on ½ MS medium supplemented with 50 mg/L hygromycin. All the experiments were carried out in the T_2_ generation. The roots of the positive transgenic plants were stained by PI solution and subcellular localization was observed with confocal microscope. All *AcYABBY* genes were also used to study the protein localization in the protoplast of pineapple [36]. 

### 4.8. Cis-Acting Elements in the Pineapple YABBY Genes Promoter region

For each pineapple *YABBY* gene, 2000 bp upstream of the translational start codon was selected from Phytozome 12 as promoter region and analyzed for presence of *cis*-acting elements using PlantCARE (http://bioinformatics.psb.ugent.be/webtools/plantcare/html/). The main *cis*-acting elements are listed in Table 2.

### 4.9. Stress Treatment

One month old tissue culture raised seedlings of pineapple (MD2 variety) were maintained under 3000 Lux light intensity and a day/night cycle of 16/8 h at 25 ± 2 °C in a controlled environment [36]. For different treatments, the seedlings were transferred to solutions containing 150 mM NaCl for salt stress, 350 mM Mannitol for osmotic stress, 100 μM ABA, 100 μM Ethephon. Cold and heat stress were performed by placing seedlings in the 4 °C and 45 °C chambers. Samples were collected at 0, 6, 12, 24 and 48h [37] after each treatment and immediately stored in liquid nitrogen.

### 4.10. Tolerance Assays under Stress Conditions

To assess phenotype under salt stress, the seeds of wild type and transgenic *Arabidopsis* lines were surface sterilized and seeded on ½ MS plates, then kept at 4 °C for 48 h in the dark before germination. About 50 seeds of each transgenic line were seeded on ½ MS medium for three days were transferred to ½ MS medium supplemented with NaCl or without NaCl and kept at 22 ± 2 °C under 16 h light/8 h dark for seven days and root length was measured. 

## 5. Conclusions

In this study, nine pineapple *YABBY* genes were identified those are preferentially expressed in different tissues. Transient expression analysis in pineapple protoplasts, tobacco leaves and *Arabidopsis* roots showed that pineapple YABBY protein were localized in the nucleus. RT-qPCR results demonstrate that *AcYABBY2*, *AcYABBY4 and AcYABBY7* are regulated by drought, NaCl, cold and heat stress. Functional analysis of *AcYABBY4* suggests that *AcYABBY4* is a negative regulator of salt stress.

## Figures and Tables

**Table 1:** Protein information of pineapple *YABBYs*, including sequenced ID, chromosome locations, isoelectric point (pI), molecular weight (MW), protein length, CDS length and exon number.

Name	Gene Locus	Chromosome Location	PI	MW (Da)	Protein Length(aa)	CDS Length(bp)	Exon
*AcYABBY1*	*Aco007606*	8	9.45	20,042.95	182	549	5
*AcYABBY2*	*Aco016279*	3	6.88	24,706.13	226	681	7
*AcYABBY3*	*Aco005138*	7	9.25	19,962.60	178	537	6
*AcYABBY4*	*Aco008751*	9	8.64	20,161.95	178	537	6
*AcYABBY5*	*Aco028479*	scaffold1235	9.05	15,073.06	136	411	5
*AcYABBY6*	*Aco002202*	4	7.71	20,872.43	188	567	6
*AcYABBY7*	*Aco003917*	15	9.05	21,453.75	190	573	7
*AcYABBY8*	*Aco026269*	12	7.01	5383.98	49	150	2
*AcYABBY9*	*Aco028478*	scaffold1235	5.82	5988.82	53	162	2

**Table 2:** Distribution of MYB, MYC and ERE *cis*-acting element in pineapple YABBY promoters.

Gene	Motif Sequence	AcYABBY1	AcYABBY2	AcYABBY3	AcYABBY4	AcYABBY5	AcYABBY6	AcYABBY7
**MYB**	TAACCA	6	5	6	9	2	4	2
	CAACAG							
	CAACCA							
**MYC**	CATTTG	4	3	4	7	8	5	5
	CATGTG							
	CAATTG							
**ERE**	ATTTCATA	3	2	0	0	1	3	0
	ATTTTAAA							

## Supplementary Materials

The following are available online at https://www.mdpi.com/1422-0067/20/23/5863/s1.

## Abbreviations

ABA	Abscisic acid;
GSDS	Gene Structure Display Server
HMM	Hidden Markov Models
MEGE	Molecular Evolutionary Genetics Analysis
MEME	Multiple Em for Motif Elicitation
RT-qPCR	Real-time Quantitative PCR
RNA-Seq	RNA sequencing
SMART	Simple Modular Architecture Research Tool
MS	Murashige and Skoog
PEG	Polyethylene glycol
WT	Wild type
GFP	Green fluorescent protein
AtYABBY	*Arabidopsis thaliana* YABBY
OsYABBY	rice (*Oryza sativa*) YABBY
AcYABBY	pineapple (*Ananas comosus*) YABBY
CDS	Coding sequence
ATG	Starting codon
FPKM	Fragments Per Kilobase Million
DAPI	4′,6-Diamidino-2-Phenylindole
MS	Murashige and Skoog
HMG	High mobility group
MYB	V-myb Myeloblastosis Viral Oncogene Homolog
MYC	Myelocytomatosis oncogenes
ERE	Ethylene Responsive Element
