# Supplementary material for: Genome-Wide Analysis of the YABBY Transcription Factor Family in Pineapple and Functional Identification of AcYABBY4 Involvement in Salt Stress
Source: Int J Mol Sci. 2019 Nov 22;20(23):5863. doi: 10.3390/ijms20235863 (PMC6929212; doi:10.3390/ijms20235863)
Supplement: Supplementary file 1 [file ijms-20-05863-s001.zip › Sup/ijms-617162 supplementary.docx]

**Figure S1.** Subcellular localization of AcYABBYs proteins in tobacco leaves. The 35S::AcYABBYs-GFP and 35S::GFP control vectors were transiently expressed in tobacco leaves and visualized by a confocal microscopy after 48-72 h of infiltration, scale bar=25μm. .

**Figure S2.** Subcellular localization of AcYABBYs proteins in *Arabidopsis* roots. 35S::AcYABBYs-GFP and 35S::GFP expressing transgenic plants were observed by confocal microscopy after five days of growth in ½MS plates, scale bar=25μm.

**Table S1.** The templates used to predict structures of YABBY proteins.

**Table S2.** The primers used in RT-qPCR.

**Table S3.** The primers used for amplifying the full-length coding sequence of the AcYABBY genes.

**Table S4.** The information of pineapple YABBY sequences in this study.
