# Supplementary material for: Genome-Wide Analysis of the YABBY Transcription Factor Family in Pineapple and Functional Identification of AcYABBY4 Involvement in Salt Stress
Source: Int J Mol Sci. 2019 Nov 22;20(23):5863. doi: 10.3390/ijms20235863 (PMC6929212; doi:10.3390/ijms20235863)
Supplement: Supplementary file 1 [file ijms-20-05863-s001.zip › Sup/Sup.pptx]

## Slide 1
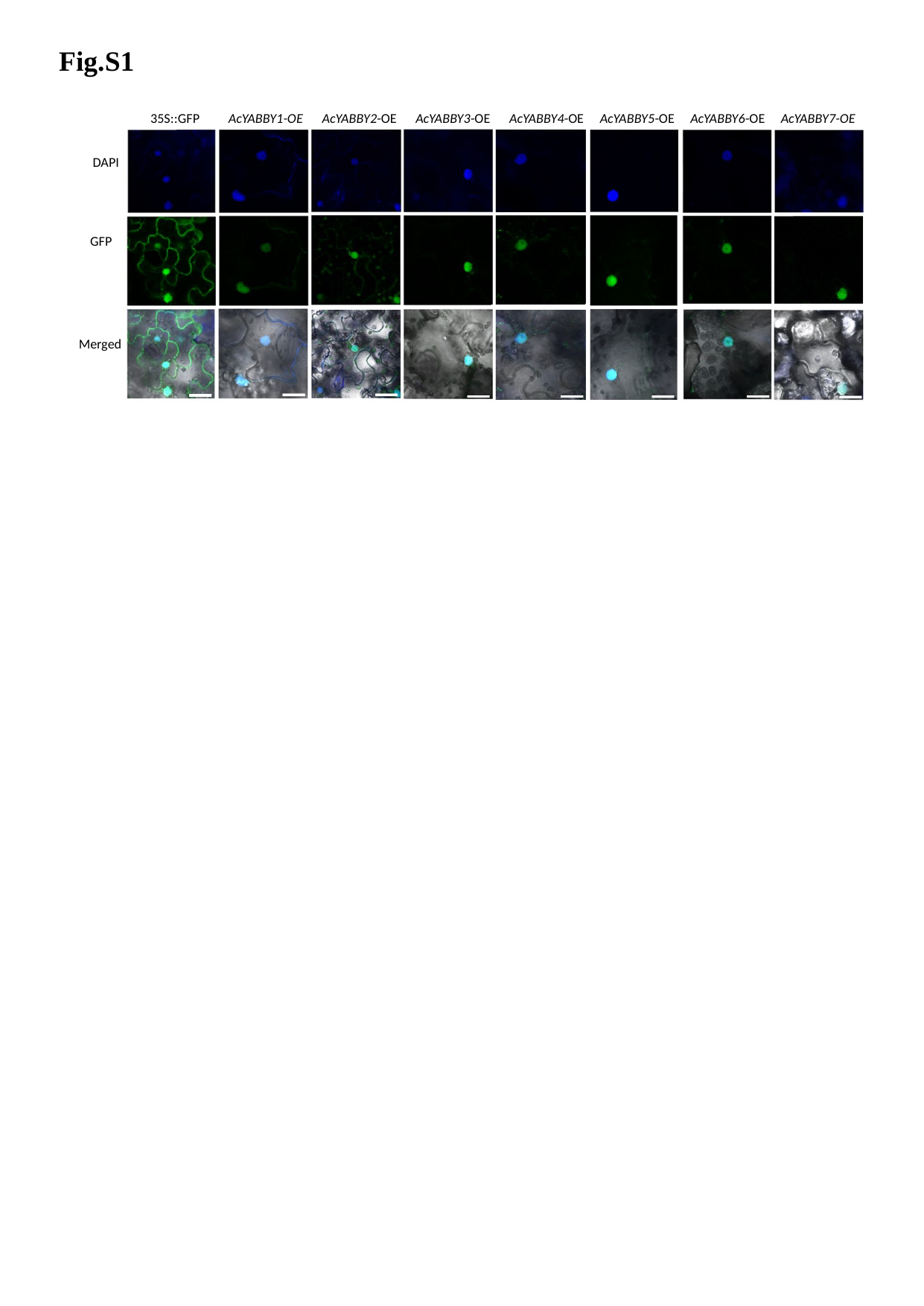

Fig.S1
 35S::GFP AcYABBY1-OE AcYABBY2-OE AcYABBY3-OE AcYABBY4-OE AcYABBY5-OE AcYABBY6-OE AcYABBY7-OE
DAPI
GFP
Merged

## Slide 2
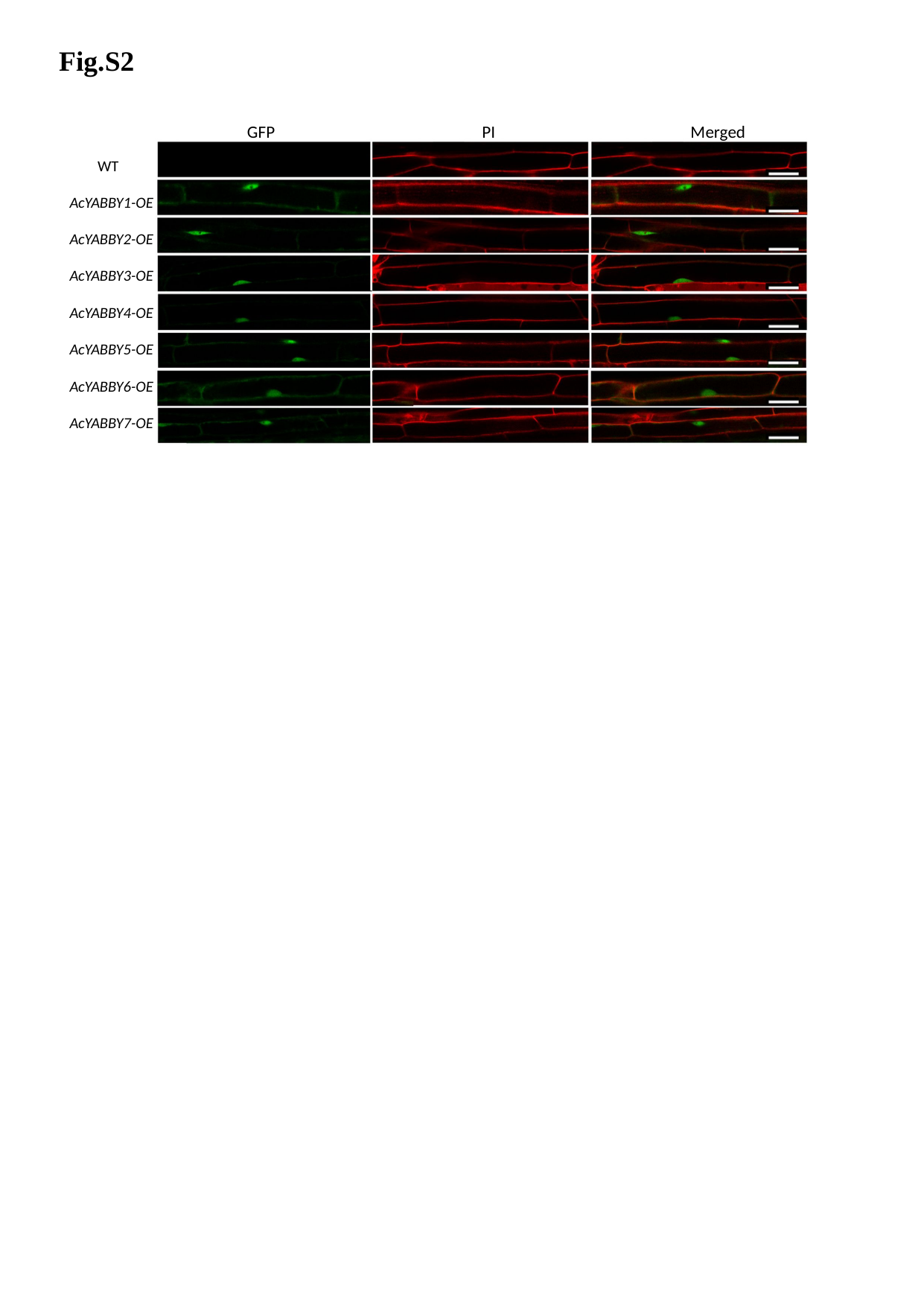

Fig.S2
 GFP PI Merged
WT
AcYABBY1-OE
AcYABBY2-OE
AcYABBY3-OE
AcYABBY4-OE
AcYABBY5-OE
AcYABBY6-OE
AcYABBY7-OE

## Slide 3
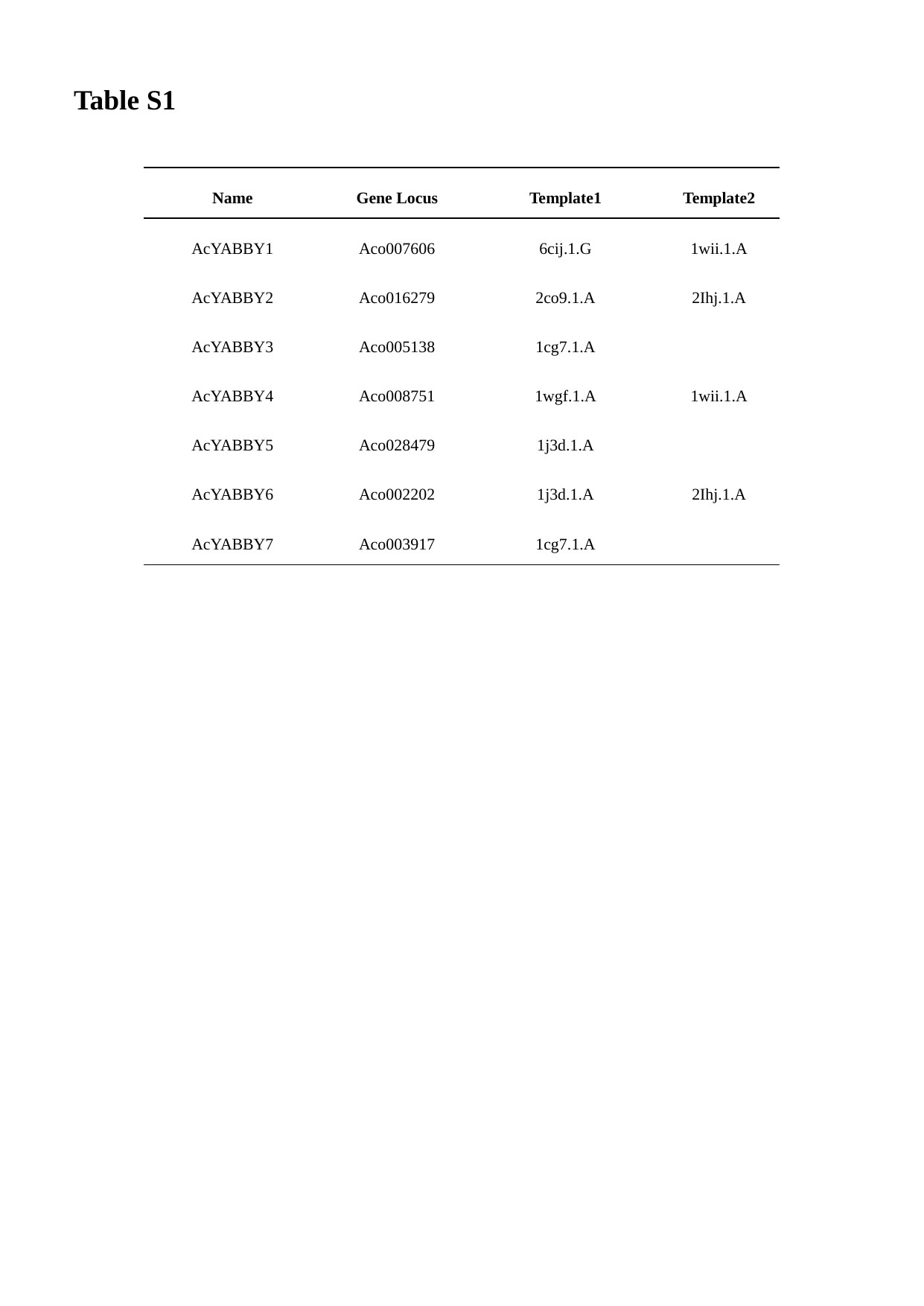

Table S1
| Name | Gene Locus | Template1 | Template2 |
| --- | --- | --- | --- |
| AcYABBY1 | Aco007606 | 6cij.1.G | 1wii.1.A |
| AcYABBY2 | Aco016279 | 2co9.1.A | 2Ihj.1.A |
| AcYABBY3 | Aco005138 | 1cg7.1.A | |
| AcYABBY4 | Aco008751 | 1wgf.1.A | 1wii.1.A |
| AcYABBY5 | Aco028479 | 1j3d.1.A | |
| AcYABBY6 | Aco002202 | 1j3d.1.A | 2Ihj.1.A |
| AcYABBY7 | Aco003917 | 1cg7.1.A | |

## Slide 4
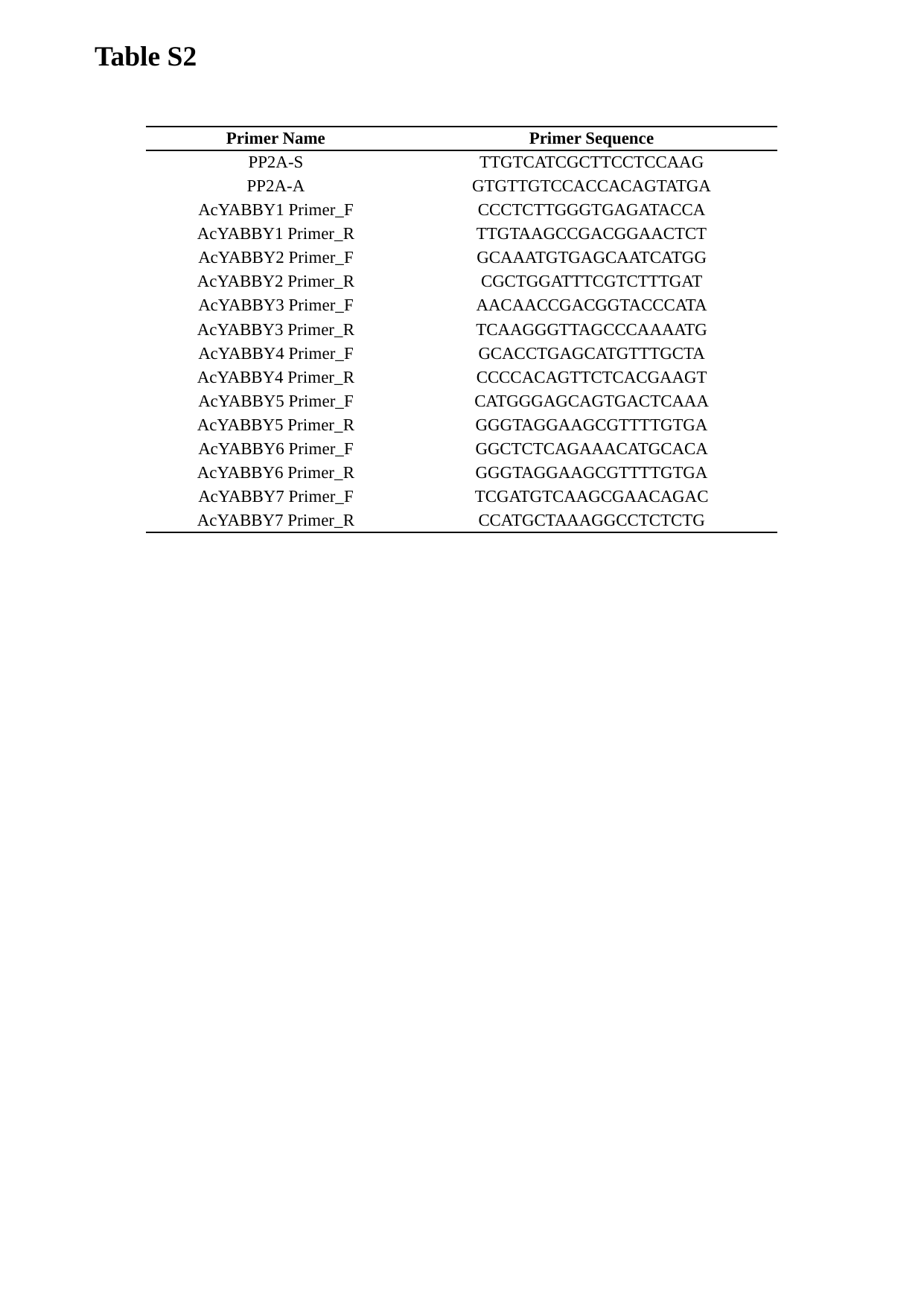

Table S2
| Primer Name | Primer Sequence |
| --- | --- |
| PP2A-S | TTGTCATCGCTTCCTCCAAG |
| PP2A-A | GTGTTGTCCACCACAGTATGA |
| AcYABBY1 Primer\_F | CCCTCTTGGGTGAGATACCA |
| AcYABBY1 Primer\_R | TTGTAAGCCGACGGAACTCT |
| AcYABBY2 Primer\_F | GCAAATGTGAGCAATCATGG |
| AcYABBY2 Primer\_R | CGCTGGATTTCGTCTTTGAT |
| AcYABBY3 Primer\_F | AACAACCGACGGTACCCATA |
| AcYABBY3 Primer\_R | TCAAGGGTTAGCCCAAAATG |
| AcYABBY4 Primer\_F | GCACCTGAGCATGTTTGCTA |
| AcYABBY4 Primer\_R | CCCCACAGTTCTCACGAAGT |
| AcYABBY5 Primer\_F | CATGGGAGCAGTGACTCAAA |
| AcYABBY5 Primer\_R | GGGTAGGAAGCGTTTTGTGA |
| AcYABBY6 Primer\_F | GGCTCTCAGAAACATGCACA |
| AcYABBY6 Primer\_R | GGGTAGGAAGCGTTTTGTGA |
| AcYABBY7 Primer\_F | TCGATGTCAAGCGAACAGAC |
| AcYABBY7 Primer\_R | CCATGCTAAAGGCCTCTCTG |

## Slide 5
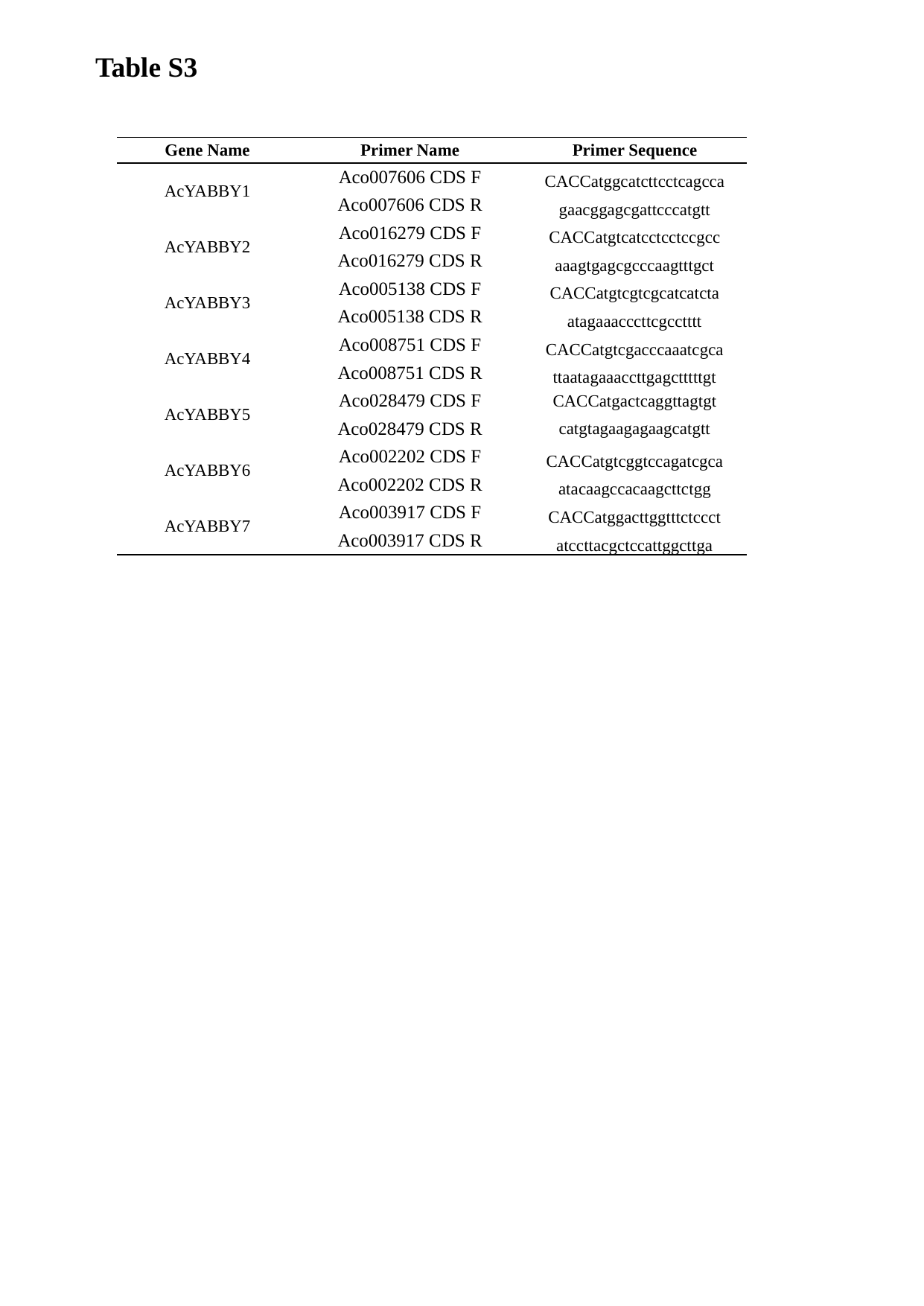

Table S3
| Gene Name | Primer Name | Primer Sequence |
| --- | --- | --- |
| AcYABBY1 | Aco007606 CDS F | CACCatggcatcttcctcagcca |
| | Aco007606 CDS R | gaacggagcgattcccatgtt |
| AcYABBY2 | Aco016279 CDS F | CACCatgtcatcctcctccgcc |
| | Aco016279 CDS R | aaagtgagcgcccaagtttgct |
| AcYABBY3 | Aco005138 CDS F | CACCatgtcgtcgcatcatcta |
| | Aco005138 CDS R | atagaaacccttcgcctttt |
| AcYABBY4 | Aco008751 CDS F | CACCatgtcgacccaaatcgca |
| | Aco008751 CDS R | ttaatagaaaccttgagctttttgt |
| AcYABBY5 | Aco028479 CDS F | CACCatgactcaggttagtgt |
| | Aco028479 CDS R | catgtagaagagaagcatgtt |
| AcYABBY6 | Aco002202 CDS F | CACCatgtcggtccagatcgca |
| | Aco002202 CDS R | atacaagccacaagcttctgg |
| AcYABBY7 | Aco003917 CDS F | CACCatggacttggtttctccct |
| | Aco003917 CDS R | atccttacgctccattggcttga |
